# Evidence of Effectiveness of Health Care Professionals Using Handheld Computers: A Scoping Review of Systematic Reviews

**DOI:** 10.2196/jmir.2530

**Published:** 2013-10-28

**Authors:** Sharon Mickan, Julie K Tilson, Helen Atherton, Nia Wyn Roberts, Carl Heneghan

**Affiliations:** ^1^Centre for Evidence Based MedicineDepartment of Primary Care Health SciencesUniversity of OxfordOxfordUnited Kingdom; ^2^Division of Biokinesiology and Physical TherapyUniversity of Southern CaliforniaLos Angeles, CAUnited States; ^3^Department of Primary Care Health SciencesUniversity of OxfordOxfordUnited Kingdom

**Keywords:** handheld computers, mobile devices, mhealth, PDA, information seeking behavior, evidence-based practice, delivery of health care, clinical practice, health technology adoption, diffusion of innovation, systematic review, evidence synthesis, documentation

## Abstract

**Background:**

Handheld computers and mobile devices provide instant access to vast amounts and types of useful information for health care professionals. Their reduced size and increased processing speed has led to rapid adoption in health care. Thus, it is important to identify whether handheld computers are actually effective in clinical practice.

**Objective:**

A scoping review of systematic reviews was designed to provide a quick overview of the documented evidence of effectiveness for health care professionals using handheld computers in their clinical work.

**Methods:**

A detailed search, sensitive for systematic reviews was applied for Cochrane, Medline, EMBASE, PsycINFO, Allied and Complementary Medicine Database (AMED), Global Health, and Cumulative Index to Nursing and Allied Health Literature (CINAHL) databases. All outcomes that demonstrated effectiveness in clinical practice were included. Classroom learning and patient use of handheld computers were excluded. Quality was assessed using the Assessment of Multiple Systematic Reviews (AMSTAR) tool. A previously published conceptual framework was used as the basis for dual data extraction. Reported outcomes were summarized according to the primary function of the handheld computer.

**Results:**

Five systematic reviews met the inclusion and quality criteria. Together, they reviewed 138 unique primary studies. Most reviewed descriptive intervention studies, where physicians, pharmacists, or medical students used personal digital assistants. Effectiveness was demonstrated across four distinct functions of handheld computers: patient documentation, patient care, information seeking, and professional work patterns. Within each of these functions, a range of positive outcomes were reported using both objective and self-report measures. The use of handheld computers improved patient documentation through more complete recording, fewer documentation errors, and increased efficiency. Handheld computers provided easy access to clinical decision support systems and patient management systems, which improved decision making for patient care. Handheld computers saved time and gave earlier access to new information. There were also reports that handheld computers enhanced work patterns and efficiency.

**Conclusions:**

This scoping review summarizes the secondary evidence for effectiveness of handheld computers and mhealth. It provides a snapshot of effective use by health care professionals across four key functions. We identified evidence to suggest that handheld computers provide easy and timely access to information and enable accurate and complete documentation. Further, they can give health care professionals instant access to evidence-based decision support and patient management systems to improve clinical decision making. Finally, there is evidence that handheld computers allow health professionals to be more efficient in their work practices. It is anticipated that this evidence will guide clinicians and managers in implementing handheld computers in clinical practice and in designing future research.

## Introduction

Handheld computing devices are changing health care delivery. Clinicians now have instant access to vast amounts of information, including x-ray results, laboratory tests, databases of primary and pre-appraised research, clinical practice guidelines, and drug reference guides. The evolution of handheld computers—smaller, more versatile, and capable of Internet connectivity—has prompted increasing usage by health care professionals. In 2003, 40% of physicians were reported to own a PDA (personal digital assistant) [1], and by 2011, over 68% of doctors in the United Kingdom were reported to own a smartphone [2]. Among medical students, 70% reported owning a smartphone in 2006 [3], increasing to 79% in 2011 [2]. There are many examples of handheld computer use in health care, including electronic prescribing, patient diagnosis and advice, patient review, practice management, reminder notifications, and eLearning.

Given the fast pace of technological innovation, the use of handheld computers has preceded definitive research about clear benefits. To date, most research has evaluated patterns of usage and adoption [4]. However, it is important to understand whether handheld computers are effective and in what settings they demonstrate improved patient care or lead to efficiencies in health care delivery. Syntheses of research evidence offer a high quality and practical way to review the existing research base. This review will scope the evidence of effectiveness across all aspects of health care practice by reviewing systematic reviews, to identify documented positive outcomes.

## Methods

### Inclusion Criteria

This review included systematic reviews published between 1992 and 2012, of all quantitative study designs, that described effective use of handheld computers by health care professionals. We defined handheld computers, consistent with the MeSH (Medical Subject Headings) term, as small, portable, and fitting in the hand. We were particularly interested in commercially available tools that health care professionals could carry with them in clinical environments. Outcomes were not pre-specified, and all aspects of demonstrated effectiveness in clinical practice were included.

### Exclusion Criteria

Systematic reviews were excluded when the focus was on the patients’ use of handheld computers, when students were learning in a classroom, and when only laptop computers were included. Systematic reviews were also excluded when they only described patterns of usage and when they focused on evaluating electronic medical records as stand-alone systems.

### Search

The following databases were searched on June 7, 2012, and December 11, 2012: Cochrane Database of Systematic Reviews (CDSR) and Database of Abstracts of Reviews of Effectiveness (DARE), Medline, EMBASE, PsycINFO, Allied and Complementary Medicine Database (AMED), Global Health, and Cumulative Index to Nursing and Allied Health Literature (CINAHL). Free-text terms and subject headings to describe handheld computers and health professionals were used as a basis of the search strategy, and these terms covered both older and newer devices (see Multimedia Appendix 1 for search strategy). Sensitive search filters developed by the Health Information Research Unit at McMaster University, Hamilton, Canada, were applied to focus the search on systematic reviews.

### Assessment of Quality

All relevant systematic reviews were independently appraised by 2 authors using the Assessment of Multiple Systematic Reviews (AMSTAR) tool [5]. This 11-item evaluation tool assesses methodological quality, presentation, and the risk of bias in systematic reviews. Systematic reviews that did not report a comprehensive search strategy or scored less than 5 out of a possible total of 11 items were excluded.

### Data Extraction

Data from included reviews were extracted independently by 2 authors to record the population studied, purpose of the review, search time frame, number and design of included studies, types of handheld computer included, and outcomes reported. A conceptual framework proposed by Free et al [6] was used to create a standardized template for data extraction. Several additional categories were created using an iterative process that involved fitting the data to amended versions of the original framework. This piloting and iterative refinement was carried out by SM and HA and continued until agreement was reached on the most appropriate categories for the data. A new template, which was used to extract objective and self-reported outcomes, summarized them according to the primary function for which the handheld computer was being used (eg, information seeking, patient data collection).

### Data Synthesis

It was expected that high levels of data heterogeneity would preclude statistical synthesis. A narrative approach was planned to summarize the evidence for effectiveness of handheld computers to support clinical practice, with respect to the primary function of the handheld computer. This involved presenting the results of each review using summary text, according to the relevant categories as determined at the data extraction stage.

## Results

### Overview

**Figure 1:**
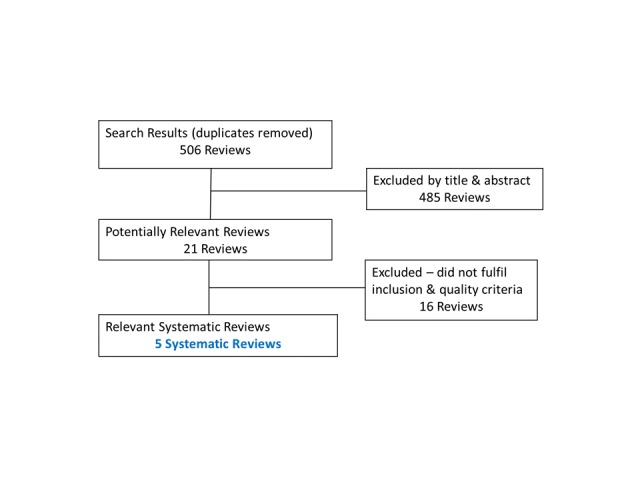
Criteria flowchart.

The initial search identified 506 systematic reviews. Of these, 21 were read for inclusion and assessed for quality using the AMSTAR checklist. Five systematic reviews met the inclusion and quality criteria (Figure 1). Included reviews scored between 5 and 8 of 11 possible points on AMSTAR (Table 1).

Table 2 describes the characteristics of the five included systematic reviews. Physicians, pharmacists, and medical students were the most common populations studied. One hundred and thirty eight unique primary studies contributed to these reviews and were published between 1995 and 2008. Of these 138 primary studies, seven were included in three of the included reviews and 19 in two of the included reviews. The lack of overlap of primary studies across these five reviews highlights the inherent heterogeneity of the field and is also reflective of the differing research questions each review addressed in relation to handheld effectiveness. Most were descriptive intervention studies, and only 8 randomized controlled trials (RCTs) were identified. All studies described handheld computers as PDAs with some having Internet connectivity and others not.

Effectiveness could be categorized across four distinct functions of handheld computers, and all five reviews identified evidence for each of the four functions (Table 3): (1) patient documentation, (2) patient care, (3) information seeking, and (4) professional work patterns. Within each function, a range of positive outcomes were reported using both objective and self-report measures.

**Table 1:** Quality evaluation of included studies.

Quality criteria	Lindquist et al, 2009	Prgomet et al, 2009	Fox et al, 2007	Kho et al, 2006	Lu et al, 2005
1. Was an a priori design provided?	1	1	1	1	1
2. Was there duplicate study selection and data extraction?	1	1	0	1	0
3. Was a comprehensive literature search performed?	1	1	1	1	1
4. Was the status of publication (ie, grey literature) used as an inclusion criterion?	1	1	0	1	1
5. Was a list of studies (included and excluded) provided?	1	1	0	0	1
6. Were the characteristics of the included studies provided?	1	1	1	0	1
7. Was the scientific quality of the included studies assessed and documented?	0	1	1	0	0
8. Was the scientific quality of the included studies used appropriately in formulating a conclusion?	0	0	1	0	0
9. Were the methods used to combine the findings of studies appropriate?	1	1	1	1	1
10. Was the likelihood of publication bias assessed?	0	0	0	0	0
11. Was the conflict of interest stated?	1	0	0	0	0
Total Score	8	8	6	5	6

**Table 2:** Descriptive characteristics of included systematic reviews.

Authors	Title	Population studied	Search time frame	Number of studies	Results
Lindquist et al, 2009	The use of the PDA among personnel and students in health care: a review	Health care professionals and students	1999-2008	48 (6 RCTs)	PDAs are used in patient care by both professionals and students with varied frequency. Their use may improve decision making, reduce number of medical errors and enhance learning.
Prgomet et al, 2009	The impact of mobile handheld technology on hospital physicians’ work practices and patient care: a systematic review	Physicians	2000-2006	13 (2 RCTs)	Handheld devices demonstrate greatest benefits in contexts where time is a critical factor; when connecting spatially distributed workers; for overcoming inadequate numbers of computers; and when data access/entry is required at point of care.
Fox et al, 2007	Use of PDAs for documentation of pharmacists’ interventions: a literature review	Pharmacists	2001-2006	12	The use of PDAs may increase the frequency and number of interventions documented by pharmacists.
Kho et al, 2006	Use of handheld computers in medical education	Medical staff and students	1995-2004	67	Handheld computers are an important and evolving part of the medical trainee’s resources in medical education and patient care.
Lu et al, 2005	A review and framework of handheld computer adoption in health care	Health care professionals	1998-2004	31	Most care providers found PDAs to be functional and useful for documentation and for access to medical references and patient data.

**Table 3:** Summary of handheld computer functions and reported effective outcomes.

Function of handheld computer	Description of function	Evidence of effectiveness (as documented in included studies)
Patient documentation	Electronic collection and documentation of patient data	More interventions recorded
		Improved documentation rates
		More accurate and detailed description of clinical findings
		More accurate diagnostic coding
		More frequent documentation of side effects
		Increased rate of electronic prescribing
		Fewer errors in discharge lists
		Improved patient identification
		Less information lost
Patient care	Access to electronic evidence based decision support systems, pharmaceutical information, transmission of investigatory images, and access to patient management systems	Reduced prescription error rates
		Fewer unsafe drug treatment decisions
		More changes in drug prescriptions
		Increased self-reported drug knowledge
		Reduced antibiotic prescription
		Decreased average length of stay
		Improved practice efficiency
		Improved diagnosis and patient care
		Shorter intervention times
		More consistent care, according to patient preferences
Information seeking	Looking for information about patients, drugs, guidelines, references, at point of care	Saves time
		More frequent access to electronic resources
		Informs patient education about medication use
		Earlier learning about new developments
Professional work patterns	Integration of handheld computers into work flows to improve efficiency and communication	Integrates well into clinical workflow
		Saves time when retrieving drug information
		Perceived efficient decision making
		Saves time in ward rounds accessing, retrieving, recording data
		More time for direct patient care
		Quicker response times and less failures to respond than with mobile phones and pagers

### Patient Documentation Outcomes

Handheld computers improved patient documentation through more complete records with fewer documentation errors and improved ease and efficiency of documentation. Pharmacists reported improved documentation rates, through recording more interventions and completing more fields [7]. Documentation using PDAs was rated significantly better than paper for detailed description of clinical findings and correct progress assessment [8]. More accurate diagnostic coding and more frequent documentation of side effects were reported [3,8]. The introduction of PDAs significantly increased the average rate of electronic prescribing from 52% to 64% (*P*=.03) [8]. Documentation with PDAs resulted in significantly fewer discrepancies in recording of neonatal patient weight in intensive care (4.4% vs 14.4% [OR 0.29, CI 0.15-0.56]) [8]. When PDAs were used to create discharge order lists, documentation errors were reduced from 22% to 8% (*P*<.05), compared to transcription from paper [1]. An electronic barcode system for identification of patients requiring blood transfusion in the hospital setting was used successfully on a PDA. There were no incidents of blood transfusion to wrong patients or wrong labeling with 41,000 samples over 3 years [9].

### Patient Care Outcomes

Improved decision making using handheld and patient management systems was a key benefit. The inclusion of specific intervention rules on handheld devices significantly reduced prescription error rates (0.23 vs 0.45; *P*<.05) [8]. Physicians using a PDA-based CDSS for prescription of nonsteroidal anti-inflammatory drugs made fewer unsafe treatment decisions [9]. Physicians reported that using a drug database developed for a PDA improved their practice efficiency, increased self-reported drug knowledge, and improved patient care [1]. There were twice as many changes in patient management when using electronic resources rather than paper resources (30% vs 18% [OR 2.00, CI 1.11-3.60]), particularly changes in drug prescription (22% vs 13% [OR 1.84, CI 0.95-3.59]) [8].

Physicians reported using PDAs loaded with locally developed guidelines and site-specific laboratory data on average 4 times per day, primarily to access laboratory data. During this 6-month prospective study, use of the PDA led to a significant decrease in antibiotics used from 1925 to 1606 daily doses per 1000 patient days (*P*=.04) and decreased the average length of patient stay by 1 day, from 7.2 to 6.2 bed days (*P*=.02) [8].

Family physicians reported that use of a PDA-based software application for cardiac stress-testing improved diagnosis and care for patients with chest pain [9]. Wireless transmission of investigatory images from PDAs to cardiologists resulted in timely and appropriate ambulance redirection and shorter intervention times [8]. Within this study, the image quality from PDAs was rated as suitable for diagnosis in all cases and identical to reference reports in most cases. Evidence-based guidelines for screening were reported as being fast and easy to use at the point of care [9]. Nurses reported that using a patient management system on a PDA made nursing care more consistent with patient preferences and improved patients’ preference achievement [9]. A patient management system available via PDAs in intensive care was described as convenient and functional, especially for patients who had long stays in hospital [9].

### Information Seeking Outcomes

Handheld computers have demonstrated effectiveness for supporting health care professionals’ information seeking needs. Where PDAs were used for self-directed learning, medical students perceived time savings of around 1 min/encounter [3]; 83% reported being better able to inform patients about medication use when looking at drug reference data [3]. When health care professionals were provided with a PDA with headlines about new books, guidelines, reviews, and medical literature, they reported learning about new developments sooner than without it [9]. Physicians accessed electronic resources via a PDA more often than paper resources (181 vs 131 episodes [OR 1.99, CI 1.41-2.80]), but average time spent in accessing them was similar (9.3 and 9.4 seconds) [8].

### Professional Work Pattern Outcomes

Handheld computers can enhance efficiency and improve patterns of work. When a PDA was used for documentation of clinical pharmacy services, 75% of users across several sites indicated that it integrated well into clinical workflow [7]. Physicians reported that PDAs enabled them to save time when retrieving information from a drug database [1]. Use of a PDA led to perceptions of more efficient decision making for patient care [9]. Physicians who utilized PDAs reported improved efficiency of their daily rounds through spending less time accessing, retrieving, and recording data, therefore freeing more time for direct patient care [1]. Median encounter time for each patient was significantly shorter when physicians used PDAs (227 vs 301 seconds) compared to paper [8]. When PDAs were compared to a mobile phone/pager for call outs, they led to shorter response times with fewer failures to respond [8,9].

## Discussion

### Principal Results

This scoping review has documented the evidence of effectiveness of handheld computers for health care professionals in four functions: patient documentation, patient care, information seeking, and professional work patterns. Across these functions, PDAs appear to provide health care professionals with timely and easy access to relevant information, facilitate accurate and complete documentation, coordinate information at the point of care, and support efficient work flows.

It is important to recognize that the pace of change of technology is faster than the rate of research production in this area. While the included systematic reviews in this review focused on evaluating the effectiveness of PDAs as handheld computers, current practice reflects the widespread use of smartphones, which were only introduced to the market in 2007 [2]. However, technology has changed steadily over time. For example, later PDAs had Internet connectivity and could run specific applications. It is therefore anticipated that the evidence for effectiveness identified in this review will, for the most part, hold true for smartphones. While the devices used may evolve quickly over time, the behaviors and actions of the clinicians using them change at a much slower rate. We can also expect that as hardware and software continue to develop, there will be enhanced and additional benefits. In future updates of this review, we would expect to see systematic reviews of smartphone use. Further, as more patients have smartphones, there are new opportunities for direct communication with health care professionals and for improved self-monitoring and disease prevention. Already, there are many available apps for patient use to enhance wellness through promoting diet and exercise and limiting smoking and alcohol use [10].

Two reviews included in this study superficially addressed issues of cost avoidance and cost savings [1,7]. While savings are likely to be of interest to managers and policymakers, there is need for better understanding of real costs. Medical students and junior doctors have expressed concern about the high costs of smartphones and medical apps [2]. Certainly, widespread implementation of continually evolving handheld computer technology in health care organizations demands economic analyses.

Similarly, the views of health care professionals need to be carefully evaluated in relation to barriers and facilitators of handheld computer use. While positive perceptions about efficiency have been documented, concerns have been raised about lack of user-friendly interfaces [7], encryption of patient data [8], and the practical issues of data crashing [7] and hardware breakage [11]. More recently, doctors have raised concerns about the impact of using smartphones in a clinical environment on the doctor-patient relationship and uncertainty about patients’ perceptions and expectations [2].

This study identifies five systematic reviews that provide evidence of the effective use of handheld computers by health care professionals, as a snapshot of current research evidence. It is anticipated that this will provide direction for clinicians and managers who may be implementing handheld computers in clinical practice and for designing future research. The clinicians of 2012 used smartphones and tablets rather than PDAs, but the lessons to be learned from the use of PDAs should not be discounted; technology has become more sophisticated but facilitates similar actions.

### Study Limitations

In order to quickly summarize the research evidence within this fairly young academic field, this scoping review included only the evidence of effectiveness reported within included systematic reviews. Reviews of systematic reviews provide a succinct overview of the field, with a focus on studies representing the highest quality of evidence synthesis [12]. While this methodology is especially useful where there is heterogeneity of study design and outcomes, it has limitations. Being removed from the primary data by two levels poses difficulties in synthesizing the resulting data. Although primary studies were checked when deemed necessary, this paper summarized only the data provided by the systematic review authors.

We recognize that neither this scoping review nor any of the included systematic reviews were able to statistically pool effectiveness data. We also acknowledge the high potential for bias associated with the predominance of low quality primary studies. It is also likely that key benefits may have been missed because of measurement limitations in primary studies. Further, it lacks a balanced evaluation of effectiveness for and against each of the functions identified. The evidence reported in this review is also subject to a significant time lag in research production. Over time, systematic reviews of smartphone use will begin to proliferate, but at the time of our search, we did not identify any that met our inclusion criteria.

### Areas for Future Research

While this review has presented evidence for better access to patient results and reductions in adverse events and hospital length of stay, there is a need to replicate and better understand these benefits. Effectiveness of handheld computers also needs to be reviewed from the perspectives of patients, health care students, and health care organizations. There is also a need to understand the mechanisms by which handheld computers support clinical practice, and this may require complementary qualitative and mixed methods studies.

### Conclusions

There is emerging evidence of effectiveness for the use of handheld computers by health care professionals across a variety of functions that support clinical practice. Handheld computers appear to provide easy and timely access to information and to support more accurate and complete documentation. They can also provide access to evidence-based decision support and patient management systems that improve clinical decision making for patient care. Finally, there is evidence that handheld computers allow health professionals to be more efficient in their work practices, thereby allowing more time for patient contact. This evidence may guide clinicians, managers, and researchers in incorporating the growing number of ever more sophisticated devices into routine clinical practice and future research. We should utilize it in assessing whether emerging devices are living up to their hype.

## Multimedia Appendix 1

Complete search strategy.

## Abbreviations

AMSTAR: Assessment of Multiple Systematic Reviews
CDSS: computer-based clinical decision support systems
OR: odds ratio
PDA: personal digital assistant
RCT: randomized controlled trial

## References

[1] Lu YC, Xiao Y, Sears A, Jacko JA (2005). A review and a framework of handheld computer adoption in healthcare. Int J Med Inform.

[2] Payne KB, Wharrad H, Watts K (2012). Smartphone and medical related App use among medical students and junior doctors in the United Kingdom (UK): a regional survey. BMC Med Inform Decis Mak.

[3] Kho A, Henderson LE, Dressler DD, Kripalani S (2006). Use of handheld computers in medical education. A systematic review. J Gen Intern Med.

[4] Garritty C, El Emam K (2006). Who's using PDAs? Estimates of PDA use by health care providers: a systematic review of surveys. J Med Internet Res.

[5] Shea BJ, Grimshaw JM, Wells GA, Boers M, Andersson N, Hamel C, Porter AC, Tugwell P, Moher D, Bouter LM (2007). Development of AMSTAR: a measurement tool to assess the methodological quality of systematic reviews. BMC Med Res Methodol.

[6] Free C, Phillips G, Felix L, Galli L, Patel V, Edwards P (2010). The effectiveness of M-health technologies for improving health and health services: a systematic review protocol. BMC Res Notes.

[7] Fox BI, Felkey BG, Berger BA, Krueger KP, Rainer RK (2007). Use of personal digital assistants for documentation of pharmacists' interventions: a literature review. Am J Health Syst Pharm.

[8] Prgomet M, Georgiou A, Westbrook JI (2009). The impact of mobile handheld technology on hospital physicians' work practices and patient care: a systematic review. J Am Med Inform Assoc.

[9] Lindquist AM, Johansson PE, Petersson GI, Saveman BI, Nilsson GC (2008). The use of the Personal Digital Assistant (PDA) among personnel and students in health care: a review. J Med Internet Res.

[10] Ozdalga E, Ozdalga A, Ahuja N (2012). The smartphone in medicine: a review of current and potential use among physicians and students. J Med Internet Res.

[11] Black AD, Car J, Pagliari C, Anandan C, Cresswell K, Bokun T, McKinstry B, Procter R, Majeed A, Sheikh A (2011). The impact of eHealth on the quality and safety of health care: a systematic overview. PLoS Med.

[12] Bastian H, Glasziou P, Chalmers I (2010). Seventy-five trials and eleven systematic reviews a day: how will we ever keep up?. PLoS Med.

