# Supplementary material for: Evidence of Effectiveness of Health Care Professionals Using Handheld Computers: A Scoping Review of Systematic Reviews
Source: J Med Internet Res. 2013 Oct 28;15(10):e212. doi: 10.2196/jmir.2530 (PMC3841346; doi:10.2196/jmir.2530)
Supplement: Supplementary file 1 [file jmir_v15i10e212_app1.pdf]

Title:

Hand-held computers - Reviews

Search summary:

We searched the Cochrane Database of Systematic Reviews and the Database of Abstracts of Reviews of Effectiveness [The Cochrane Library, Wiley] (Issue 11, 2012), Medline [OvidSP] (1946 – , In process), Embase [OvidSP] (1974 –), CINAHL [EbscoHOST] (1980 –), PsycINFO [OvidSP] (1967 -), Global Health [OvidSP] (1973 -) and AMED [OvidSP] (1985 -). Searches were conducted on 7th June 2012 & 11th December 2012

Search methods:

**Database name:**	**Interface:**	**Year range:**	**Hits:**
AMED	OvidSP	1985 -	20
CINAHL	EbscoHOST	1980 –	29
CDSR & DARE	Cochrane Library, Wiley	Issue 11. 2012	12
Embase	OvidSP	1974 –	312
Global Health	OvidSP	1973 -	4
Medline	OvidSP	1946 - , In process	286
PsycINFO	OvidSP	1967 –	40

Total number of records retrieved = 703
Number of duplicates removed = 197
Final total = 506

Limits:

Publication type: Systematic reviews

Search strategies:

AMED (OvidSP) [1985 -] – 7th June 2012 & 11th December 2012

1	exp health personnel/	3858
2	clinician*1.ti,ab.	3959
3	(clinical adj3 (staff or personnel or officer*1 or worker*1)).ti,ab.	135
4	(medical adj3 (staff or personnel or officer*1 or worker*1 or professional*)).ti,ab.	487
5	((health or healthcare) adj3 (staff or personnel or officer* or worker*1 or professional*1)).ti,ab.	3293
6	(physician*1 or doctor*1 or surgeon*1).ti,ab.	8036
7	nurse*1.ti,ab.	4330
8	practitioner*1.ti,ab.	4558
9	(physical therapist*1 or physiotherapist*1).ti,ab.	4024
10	rehabilitation therapist*1.ti,ab.	40
11	occupational therapist*1.ti,ab.	2863
12	((speech or language) adj3 therapist*).ti,ab.	232
13	1 or 2 or 3 or 4 or 5 or 6 or 7 or 8 or 9 or 10 or 11 or 12	28450
14	(mhealth* or m-health* or mobile health*).ti,ab.	2
15	(handheld adj3 (device* or computer* or technolog* or PC)).ti,ab.	10
16	(hand-held adj3 (device* or computer* or technolog* or PC)).ti,ab.	19
17	(tablet adj3 (device* or computer* or technolog* or PC)).ti,ab.	6

18	(slate adj3 tablet*).ti,ab.	1
19	(android adj3 tablet*).ti,ab.	0
20	(palm*1 adj3 (device* or computer* or pc)).ti,ab.	10
21	(pocket* adj3 (device* or computer* or pc)).ti,ab.	10
22	(mobile adj3 (computer* or PC)).ti,ab.	5
23	(portable adj3 (computer* or PC)).ti,ab.	13
24	(palm pilot* or palmpilot*).ti,ab.	1
25	(smartbook* or smart-book*).ti,ab.	0
26	ipad*.ti,ab.	3
27	i-pad*.ti,ab.	0
28	galaxy tab.ti,ab.	0
29	kindle fire.ti,ab.	0
30	playbook.ti,ab.	0
31	((touchscreen or touch screen) adj3 (computer* or PC or technolog* or device*)).ti,ab.	9
32	(smartphone* or smart phone*).ti,ab.	9
33	iphone*.ti,ab.	0

34	i-phone*.ti,ab.	0
35	(blackberry or black-berry).ti,ab.	8
36	(android adj3 (phone* or cellphone* or telephone* or device* or mobile*)).ti,ab.	1
37	(google adj3 (phone* or cellphone* or telephone* or device* or mobile*)).ti,ab.	0
38	(nexus one adj3 (phone* or cellphone* or telephone* or device* or mobile*)).ti,ab.	0
39	(google adj3 android).ti,ab.	0
40	(ipod touch or i-pod touch).ti,ab.	1
41	((touchscreen or touch screen) adj3 (phone* or cellphone* or telephone*)).ti,ab.	0
42	apps.ti.	0
43	or/14-42	101
44	((cell or cellular) adj3 (phone* or telephone*)).ti,ab.	30
45	cellphone*.ti,ab.	3
46	(mobile adj3 (phone* or telephone* or device* or technolog*)).ti,ab.	49
47	digital assistant*.ti,ab.	21
48	pda*.ti,ab.	44
49	44 or 45 or 46 or 47 or 48	123

50	internet.ti,ab.	620
51	(online or on-line).ti,ab.	2690
52	(web* or www).ti,ab.	900
53	(wireless or wifi or wi-fi or wap).ti,ab.	53
54	(bluetooth or blue tooth).ti,ab.	2
55	50 or 51 or 52 or 53 or 54	3917
56	49 and 55	15
57	43 or 56	116
58	13 and 57	20

CINAHL (EBSCOHost) (1980 -) – 7th June 2012 & 11th December 2012

S63	S59 AND S62
S62	S60 OR S61
S61	PT review OR PT systematic review
S60	TI meta-analysis OR AB meta-analysis OR MW meta-analysis
S59	S14 and S58
S5	S43 or S57

8	
S57	S49 and S56
S56	S50 or S51 or S52 or S53 or S54 or S55
S55	TI (bluetooth or blue tooth) OR AB (bluetooth or blue tooth)
S54	TI (wireless or wifi or wi-fi or wap) OR AB (wireless or wifi or wi-fi or wap)
S53	TI (web* or www) OR AB (web* or www)
S52	TI (online or on-line) OR AB (online or on-line)
S51	TI internet OR AB internet
S50	(MH "Internet")
S49	S44 or S45 or S46 or S47 or S48
S48	TI (digital assistant* or pda) OR AB (digital assistant* or pda)
S47	TI (mobile n3 phone* or mobile n3 telephone* or mobile n3 device* or mobile n3 technolog*) OR AB (mobile n3 phone* or mobile n3 telephone* or mobile n3 device* or mobile n3 technolog*)
S46	TI cellphone* OR AB cellphone*
S45	TI (cell n3 phone* or cell n3 telephone* or cellular n3 phone* or cellular n3 telephone*) OR AB (cell n3 phone* or cell n3 telephone* or cellular n3 phone* or cellular n3 telephone*)
S44	(MH "Telephone") AND (MH "Wireless Communications")
S43	S15 or S16 or S17 or S18 or S19 or S20 or S21 or S22 or S23 or S24 or S25 or S26 or S27 or S28 or S29 or S30 or S31 or S32 or S33 or S34 or S35 or S36 or S37 or S38 or S39 or S40 or S41 or S42
S4	TI apps OR AB apps

2	
S41	TI (touch-screen n3 phone* or touch-screen n3 telephone* or touch-screen n3 cellphone* or touch-screen n3 device* or touch-screen n3 mobile) OR AB (touch-screen n3 phone* or touch-screen n3 telephone* or touch-screen n3 cellphone* or touch-screen n3 device* or touch-screen n3 mobile)
S40	TI (touchscreen n3 phone* or touchscreen n3 telephone* or touchscreen n3 cellphone* or touchscreen n3 device* or touchscreen n3 mobile) OR AB (touchscreen n3 phone* or touchscreen n3 telephone* or touchscreen n3 cellphone* or touchscreen n3 device* or touchscreen n3 mobile)
S39	TI (ipod touch or i-pod touch) OR AB (ipod touch or i-pod touch)
S38	TI google n3 android OR AB google n3 android
S37	TI (nexus one n3 phone* or nexus one n3 telephone* or nexus one n3 cellphone* or nexus one n3 device* or nexus one n3 mobile) OR AB (nexus one n3 phone* or nexus one n3 telephone* or nexus one n3 cellphone* or nexus one n3 device* or nexus one n3 mobile)
S36	TI (google n3 phone* or google n3 telephone* or google n3 cellphone* or google n3 device* or google n3 mobile) OR AB (google n3 phone* or google n3 telephone* or google n3 cellphone* or google n3 device* or google n3 mobile)
S35	TI (android n3 phone* or android n3 telephone* or android n3 cellphone* or android n3 device* or android n3 mobile) OR AB (android n3 phone* or android n3 telephone* or android n3 cellphone* or android n3 device* or android n3 mobile)
S34	TI (blackberry or black-berry) OR AB (blackberry or black-berry)
S33	TI (iphone* or i-phone*) OR AB (iphone* or i-phone*)
S32	TI (touch-screen n3 computer* or touch-screen n3 pc or touch-screen n3 technolog* or touch-screen n3 device*) OR AB (touch-screen n3 computer* or touch-screen n3 pc or touch-screen n3 technolog* or touch-screen n3 device*)
S31	TI (touchscreen n3 computer* or touchscreen n3 pc or touchscreen n3 technolog* or touchscreen n3 device*) OR AB (touchscreen n3 computer* or touchscreen n3 pc or touchscreen n3 technolog* or touchscreen n3 device*)
S3	TI kindle fire OR AB kindle fire

0	
S29	TI galaxy tab OR AB galaxy tab
S28	TI (ipad* or i-pad*) OR AB (ipad* or i-pad*)
S27	TI (smartbook* or smart-book*) OR AB (smartbook* or smart-book*)
S26	TI (palm pilot* or palmpilot) OR AB (palm pilot* or palmpilot)
S25	TI (portable n3 computer* or portable n3 pc) OR AB (portable n3 computer* or portable n3 pc)
S24	TI (mobile computer* or mobile pc) OR AB (mobile computer* or mobile pc)
S23	TI (pocket n3 device* or pocket n3 computer* or pocket n3 pc) OR AB (pocket n3 device* or pocket n3 computer* or pocket n3 pc)
S22	TI (palm* n3 device* or palm* n3 computer* or palm* n3 pc) OR AB (palm* n3 device* or palm* n3 computer* or palm* n3 pc)
S21	TI android n3 tablet* OR AB android n3 tablet*
S20	TI slate n3 tablet* OR AB slate n3 tablet*
S19	TI (tablet n3 device* or tablet n3 computer* or tablet technolog* or tablet n3 pc) OR AB (tablet n3 device* or tablet n3 computer* or tablet technolog* or tablet n3 pc)
S18	TI (hand-held n3 device* or hand-held n3 computer* or hand-held technolog* or hand-held n3 pc) OR AB (hand-held n3 device* or hand-held n3 computer* or hand-held technolog* or hand-held n3 pc)
S17	TI (handheld n3 device* or handheld n3 computer* or handheld technolog* or handheld n3 pc) OR AB (handheld n3 device* or handheld n3 computer* or handheld technolog* or handheld n3 pc)
S16	TI (mhealth* or m-health* or mobile health*) OR AB (mhealth* or m-health* or mobile health*)
S15	(MH "Computers, Hand-Held")
S1	S1 or S2 or S3 or S4 or S5 or S6 or S7 or S8 or S9 or S10 or S11 or S12 or S13

4	
S13	TI (speech therapist* or language therapist*) OR AB (speech therapist* or language therapist*)
S12	TI occupational therapist* OR AB occupational therapist*
S11	TI rehabilitation therapist* OR AB rehabilitation therapist*
S10	TI (physical therapist* or physiotherapist*) OR AB (physical therapist* or physiotherapist*)
S9	TI practitioner* OR AB practitioner*
S8	TI nurse* OR AB nurse*
S7	TI (physician* or doctor* or surgeon*) OR AB (physician* or doctor* or surgeon*)
S6	TI (healthcare n3 staff or healthcare n3 personnel or healthcare n3 officer* or healthcare n3 worker* or healthcare n3 professional) OR AB (healthcare n3 staff or healthcare n3 personnel or healthcare n3 officer* or healthcare n3 worker* or healthcare n3 professional)
S5	TI (health n3 staff or health n3 personnel or health n3 officer* or health n3 worker* or health n3 professional) OR AB (health n3 staff or health n3 personnel or health n3 officer* or health n3 worker* or health n3 professional)
S4	TI (medical n3 staff or medical n3 personnel or medical n3 officer* or medical n3 worker* or medical n3 professional) OR AB (medical n3 staff or medical n3 personnel or medical n3 officer* or medical n3 worker* or medical n3 professional)
S3	TI (clinical n3 staff or clinical n3 personnel or clinical n3 officer* or clinical n3 worker*) OR AB (clinical n3 staff or clinical n3 personnel or clinical n3 officer* or clinical n3 worker*)
S2	TI (clinician or clinicians) OR AB (clinician or clinicians)
S1	(MH "Health Personnel+")

Cochrane Database of Systematic Reviews & Database of Abstracts of Reviews of Effectiveness (Cochrane Library, Wiley) [Issue 11, 2012] - 7th June 2012 & 11th December 2012

#1 MeSH descriptor Health Personnel explode all trees 4532
#2 MeSH descriptor Health Occupations explode all trees 13691
#3 (clinician*):ti,ab,kw 5215
#4 (clinical near3 (staff or personnel or officer* or worker*)):ti,ab,kw 406
#5 (medical near3 (staff or personnel or officer* or worker* or professional*)):ti,ab,kw 914
#6 ((health or healthcare) near3 (staff or personnel or officer* or worker* or professional*)):ti,ab,kw 4354
#7 (physician* or doctor* or surgeon*):ti,ab,kw 19262
#8 (nurse*):ti,ab,kw 7850
#9 (practitioner*):ti,ab,kw 4332
#10 (physical therapist* or physiotherapist*):ti,ab,kw 1044
#11 (rehabilitation therapist*):ti,ab,kw 227
#12 (occupational therapist*):ti,ab,kw 220
#13 ((speech or language) near3 therapist*):ti,ab,kw 53
#14 (#1 OR #2 OR #3 OR #4 OR #5 OR #6 OR #7 OR #8 OR #9 OR #10 OR #11 OR #12 OR #13) 46408
#15 MeSH descriptor Computers, Handheld explode all trees 103
#16 (mhealth* or m-health* or mobile health*):ti,ab,kw 285
#17 (handheld near3 (device* or computer* or technolog* or PC)):ti,ab,kw 152
#18 (hand-held near3 (device* or computer* or technolog* or PC)):ti,ab,kw 80
#19 (tablet near3 (device* or computer* or technolog* or PC)):ti,ab,kw 36
#20 (slate near3 tablet*):ti,ab,kw 0
#21 (android near3 tablet*):ti,ab,kw 0
#22 (palm* near3 (device* or computer* or pc)):ti,ab,kw 25
#23 (pocket* near3 (device* or computer* or pc)):ti,ab,kw 27
#24 (mobile near3 (computer* or PC)):ti,ab,kw 10
#25 (portable near3 (computer* or PC)):ti,ab,kw 17
#26 (palm pilot* or palmpilot*):ti,ab,kw 9
#27 (smartbook* or smart-book*):ti,ab,kw 0
#28 (ipad*):ti,ab,kw 0
#29 (i-pad*):ti,ab,kw 0
#30 (galaxy tab):ti,ab,kw 0
#31 (kindle fire):ti,ab,kw 0
#32 (playbook):ti,ab,kw 0
#33 ((touchscreen or touch screen) near3 (computer* or PC or technolog* or

device*)):ti,ab,kw 48
#34 (smartphone* or smart phone*):ti,ab,kw 7
#35 (iphone* or i-phone*):ti,ab,kw 0
#36 (blackberry or black-berry):ti,ab,kw 6
#37 (android near3 (phone* or cellphone* or telephone* or device* or mobile*)):ti,ab,kw 0
#38 (google near3 (phone* or cellphone* or telephone* or device* or mobile*)):ti,ab,kw 0
#39 (nexus one near3 (phone* or cellphone* or telephone* or device* or mobile*)):ti,ab,kw 0
#40 (google near3 android):ti,ab,kw 0
#41 (ipod touch or i-pod touch):ti,ab,kw 1
#42 ((touchscreen or touch screen) near3 (phone* or cellphone* or telephone*)):ti,ab,kw 2
#43 (apps):ti,ab,kw 9
#44 (#15 OR #16 OR #17 OR #18 OR #19 OR #20 OR #21 OR #23 OR #24 OR #25 OR #26 OR #27 OR #28 OR #29 OR #30 OR #31 OR #32 OR #33 OR #34 OR #35 OR #36 OR #37 OR #38 OR #39 OR #40 OR #41 OR #42 OR #43) 642
#45 MeSH descriptor Cellular Phone explode all trees 186
#46 ((cell or cellular) near3 (phone* or telephone*)):ti,ab,kw 243
#47 (cellphone*):ti,ab,kw 3
#48 (mobile near3 (phone* or telephone* or device* or technolog*)):ti,ab,kw 212
#49 (digital assistant*):ti,ab,kw 81
#50 (pda*):ti,ab,kw 286
#51 (#45 OR #46 OR #47 OR #48 OR #49 OR #50) 633
#52 MeSH descriptor Internet explode all trees 1191
#53 MeSH descriptor Wireless Technology explode all trees 4
#54 (internet):ti,ab,kw 2492
#55 (online or on-line):ti,ab,kw 1603
#56 (web* or www):ti,ab,kw 3613
#57 (wireless or wifi or wi-fi or wap):ti,ab,kw 107
#58 (bluetooth or blue tooth):ti,ab,kw 129
#59 (#52 OR #53 OR #54 OR #55 OR #56 OR #57 OR #58) 6228
#60 (#51 AND #59) 90
#61 (#44 OR #60) 703
#62 (#14 AND #61)

Embase (OvidSP) (1974 -) – 7th June 2012 & 11th December 2012

1	exp health care personnel/	772226
2	clinician*1.ti,ab.	143842
3	(clinical adj3 (staff or personnel or officer*1 or worker*1)).ti,ab.	5248
4	(medical adj3 (staff or personnel or officer*1 or worker*1 or professional*)).ti,ab.	31885
5	((health or healthcare) adj3 (staff or personnel or officer* or worker*1 or professional*1)).ti,ab.	100264
6	(physician*1 or doctor*1 or surgeon*1).ti,ab.	575966
7	nurse*1.ti,ab.	193433
8	practitioner*1.ti,ab.	111348
9	(physical therapist*1 or physiotherapist*1).ti,ab.	10507
10	rehabilitation therapist*1.ti,ab.	161
11	occupational therapist*1.ti,ab.	5354
12	((speech or language) adj3 therapist*).ti,ab.	1885
13	1 or 2 or 3 or 4 or 5 or 6 or 7 or 8 or 9 or 10 or 11 or 12	1479023
14	(mhealth* or m-health* or mobile health*).ti,ab.	404
15	(handheld adj3 (device* or computer* or technolog* or PC)).ti,ab.	1019
16	(hand-held adj3 (device* or computer* or technolog* or PC)).ti,ab.	849
17	(tablet adj3 (device* or computer* or technolog* or PC)).ti,ab.	436

18	(slate adj3 tablet*).ti,ab.	3
19	(android adj3 tablet*).ti,ab.	6
20	(palm*1 adj3 (device* or computer* or pc)).ti,ab.	140
21	(pocket* adj3 (device* or computer* or pc)).ti,ab.	428
22	(mobile adj3 (computer* or PC)).ti,ab.	247
23	(portable adj3 (computer* or PC)).ti,ab.	414
24	(palm pilot* or palmpilot*).ti,ab.	56
25	(smartbook* or smart-book*).ti,ab.	1
26	ipad*.ti,ab.	349
27	i-pad*.ti,ab.	30
28	galaxy tab.ti,ab.	0
29	kindle fire.ti,ab.	0
30	playbook.ti,ab.	27
31	((touchscreen or touch screen) adj3 (computer* or PC or technolog* or device*)).ti,ab.	357
32	(smartphone* or smart phone*).ti,ab.	588
33	iphone*.ti,ab.	235

34	i-phone*.ti,ab.	17
35	(blackberry or black-berry).ti,ab.	330
36	(android adj3 (phone* or cellphone* or telephone* or device* or mobile*)).ti,ab.	18
37	(google adj3 (phone* or cellphone* or telephone* or device* or mobile*)).ti,ab.	7
38	(nexus one adj3 (phone* or cellphone* or telephone* or device* or mobile*)).ti,ab.	0
39	(google adj3 android).ti,ab.	4
40	(ipod touch or i-pod touch).ti,ab.	44
41	((touchscreen or touch screen) adj3 (phone* or cellphone* or telephone*)).ti,ab.	7
42	apps.ti.	90
43	or/14-42	5495
44	personal digital assistant/ or mobile phone/	5979
45	((cell or cellular) adj3 (phone* or telephone*)).ti,ab.	2145
46	cellphone*.ti,ab.	75
47	(mobile adj3 (phone* or telephone* or device* or technolog*)).ti,ab.	4193
48	digital assistant*.ti,ab.	1009
49	pda*.ti,ab.	9774

50	44 or 45 or 46 or 47 or 48 or 49	17756
51	Internet/	66218
52	internet.ti,ab.	31508
53	(online or on-line).ti,ab.	61971
54	(web* or www).ti,ab.	73292
55	(wireless or wifi or wi-fi or wap).ti,ab.	5777
56	(bluetooth or blue tooth).ti,ab.	301
57	51 or 52 or 53 or 54 or 55 or 56	181808
58	50 and 57	2363
59	43 or 58	7437
60	13 and 59	2176
61	medline.tw.	58134
62	meta-analys*.mp.	96408
63	search*.tw.	253490
64	review.pt.	1918292
65	61 or 62 or 63 or 64	2149928

66	60 and 65	312
67	2011*.dp,em,yr.	1401058
68	66 and 67	39

Global Health (OvidSP) (1973 -) – 7th June 2012 & 11th December 2012

1	exp health care workers/	24423
2	clinician*1.ti,ab.	9751
3	(clinical adj3 (staff or personnel or officer*1 or worker*1)).ti,ab.	552
4	(medical adj3 (staff or personnel or officer*1 or worker*1 or professional*)).ti,ab.	3745
5	((health or healthcare) adj3 (staff or personnel or officer* or worker*1 or professional*1)).ti,ab.	21430
6	(physician*1 or doctor*1 or surgeon*1).ti,ab.	30739
7	nurse*1.ti,ab.	9889
8	practitioner*1.ti,ab.	11151
9	(physical therapist*1 or physiotherapist*1).ti,ab.	265
10	rehabilitation therapist*1.ti,ab.	5
11	occupational therapist*1.ti,ab.	76
12	((speech or language) adj3 therapist*).ti,ab.	33
1	1 or 2 or 3 or 4 or 5 or 6 or 7 or 8 or 9 or 10 or 11 or 12	8056

3		4
14	(mhealth* or m-health* or mobile health*).ti,ab.	88
15	(handheld adj3 (device* or computer* or technolog* or PC)).ti,ab.	79
16	(hand-held adj3 (device* or computer* or technolog* or PC)).ti,ab.	54
17	(tablet adj3 (device* or computer* or technolog* or PC)).ti,ab.	10
18	(slate adj3 tablet*).ti,ab.	0
19	(android adj3 tablet*).ti,ab.	0
20	(palm*1 adj3 (device* or computer* or pc)).ti,ab.	9
21	(pocket* adj3 (device* or computer* or pc)).ti,ab.	13
22	(mobile adj3 (computer* or PC)).ti,ab.	23
23	(portable adj3 (computer* or PC)).ti,ab.	17
24	(palm pilot* or palmpilot*).ti,ab.	1
25	(smartbook* or smart-book*).ti,ab.	0
26	ipad*.ti,ab.	23
27	i-pad*.ti,ab.	2
28	galaxy tab.ti,ab.	0
2	kindle fire.ti,ab.	0

9		
30	playbook.ti,ab.	5
31	((touchscreen or touch screen) adj3 (computer* or PC or technolog* or device*)).ti,ab.	25
32	(smartphone* or smart phone*).ti,ab.	23
33	iphone*.ti,ab.	7
34	i-phone*.ti,ab.	1
35	(blackberry or black-berry).ti,ab.	205
36	(android adj3 (phone* or cellphone* or telephone* or device* or mobile*)).ti,ab.	0
37	(google adj3 (phone* or cellphone* or telephone* or device* or mobile*)).ti,ab.	0
38	(nexus one adj3 (phone* or cellphone* or telephone* or device* or mobile*)).ti,ab.	0
39	(google adj3 android).ti,ab.	0
40	(ipod touch or i-pod touch).ti,ab.	2
41	((touchscreen or touch screen) adj3 (phone* or cellphone* or telephone*)).ti,ab.	0
42	apps.ti.	6
43	or/14-42	568
44	((cell or cellular) adj3 (phone* or telephone*)).ti,ab.	295
4	cellphone*.ti,ab.	9

5		
46	(mobile adj3 (phone* or telephone* or device* or technolog*)).ti,ab.	633
47	digital assistant*.ti,ab.	108
48	pda*.ti,ab.	838
49	44 or 45 or 46 or 47 or 48	1742
50	internet.ti,ab.	3881
51	(online or on-line).ti,ab.	5145
52	(web* or www).ti,ab.	7140
53	(wireless or wifi or wi-fi or wap).ti,ab.	212
54	(bluetooth or blue tooth).ti,ab.	11
55	50 or 51 or 52 or 53 or 54	14416
56	49 and 55	210
57	43 or 56	758
58	13 and 57	100
59	meta-analysis.tw.	8355
60	search*.tw.	24165
6	systematic review.tw.	6685

1		
62	59 or 60 or 61	31276
63	58 and 62	4

Medline (OvidSP) (1946 - , In process) – 7th June 2012 & 11th December 2012

1	exp Health Personnel/	348969
2	exp health occupations/	1265460
3	clinician*1.ti,ab.	111946
4	(clinical adj3 (staff or personnel or officer*1 or worker*1)).ti,ab.	3987
5	(medical adj3 (staff or personnel or officer*1 or worker*1 or professional*)).ti,ab.	24979
6	((health or healthcare) adj3 (staff or personnel or officer* or worker*1 or professional*1)).ti,ab.	83154
7	(physician*1 or doctor*1 or surgeon*1).ti,ab.	438274
8	nurse*1.ti,ab.	175531
9	practitioner*1.ti,ab.	90502
10	(physical therapist*1 or physiotherapist*1).ti,ab.	7001
11	rehabilitation therapist*1.ti,ab.	104
12	occupational therapist*1.ti,ab.	3976
13	((speech or language) adj3 therapist*).ti,ab.	1253
14	1 or 2 or 3 or 4 or 5 or 6 or 7 or 8 or 9 or 10 or 11 or 12 or 13	2044473

15	Computers, Handheld/	1927
16	(mhealth* or m-health* or mobile health*).ti,ab.	411
17	(handheld adj3 (device* or computer* or technolog* or PC)).ti,ab.	846
18	(hand-held adj3 (device* or computer* or technolog* or PC)).ti,ab.	693
19	(tablet adj3 (device* or computer* or technolog* or PC)).ti,ab.	277
20	(slate adj3 tablet*).ti,ab.	3
21	(android adj3 tablet*).ti,ab.	1
22	(palm*1 adj3 (device* or computer* or pc)).ti,ab.	125
23	(pocket* adj3 (device* or computer* or pc)).ti,ab.	297
24	(mobile adj3 (computer* or PC)).ti,ab.	192
25	(portable adj3 (computer* or PC)).ti,ab.	343
26	(palm pilot* or palmpilot*).ti,ab.	46
27	(smartbook* or smart-book*).ti,ab.	2
28	ipad*.ti,ab.	240
29	i-pad*.ti,ab.	19
30	galaxy tab.ti,ab.	0

31	kindle fire.ti,ab.	0
32	playbook.ti,ab.	29
33	((touchscreen or touch screen) adj3 (computer* or PC or technolog* or device*)).ti,ab.	261
34	(smartphone* or smart phone*).ti,ab.	430
35	iphone*.ti,ab.	129
36	i-phone*.ti,ab.	5
37	(blackberry or black-berry).ti,ab.	258
38	(android adj3 (phone* or cellphone* or telephone* or device* or mobile*)).ti,ab.	15
39	(google adj3 (phone* or cellphone* or telephone* or device* or mobile*)).ti,ab.	3
40	(nexus one adj3 (phone* or cellphone* or telephone* or device* or mobile*)).ti,ab.	0
41	(google adj3 android).ti,ab.	2
42	(ipod touch or i-pod touch).ti,ab.	30
43	((touchscreen or touch screen) adj3 (phone* or cellphone* or telephone*)).ti,ab.	4
44	apps.ti.	80
45	or/15-44	5635
46	Cellular Phone/	3339

47	((cell or cellular) adj3 (phone* or telephone*)).ti,ab.	1724
48	cellphone*.ti,ab.	44
49	(mobile adj3 (phone* or telephone* or device* or technolog*)).ti,ab.	3474
50	digital assistant*.ti,ab.	853
51	pda*.ti,ab.	7026
52	46 or 47 or 48 or 49 or 50 or 51	13046
53	exp Internet/	43923
54	Software/	71363
55	Wireless Technology/	464
56	internet.ti,ab.	24539
57	(online or on-line).ti,ab.	48022
58	(web* or www).ti,ab.	60223
59	(wireless or wifi or wi-fi or wap).ti,ab.	5212
60	(bluetooth or blue tooth).ti,ab.	248
61	53 or 54 or 55 or 56 or 57 or 58 or 59 or 60	200166
62	52 and 61	2009

63	45 or 62	6971
64	14 and 63	2598
65	meta-analysis.mp,pt.	61991
66	review.pt.	1759271
67	search*.tw.	208914
68	65 or 66 or 67	1926581
69	64 and 68	286
70	2012*.dp,yr,ed.	1327144
71	69 and 70	55

PsycINFO (OvidSP) (1967 -) – 7th June 2012 & 11th December 2012

1	exp health personnel/ or exp therapists/ or clinicians/	102387
2	clinician*1.ti,ab.	51967
3	(clinical adj3 (staff or personnel or officer*1 or worker*1)).ti,ab.	1897
4	(medical adj3 (staff or personnel or officer*1 or worker*1 or professional*)).ti,ab.	5341
5	((health or healthcare) adj3 (staff or personnel or officer* or worker*1 or professional*1)).ti,ab.	34941

6	(physician*1 or doctor*1 or surgeon*1).ti,ab.	54952
7	nurse*1.ti,ab.	35733
8	practitioner*1.ti,ab.	52500
9	(physical therapist*1 or physiotherapist*1).ti,ab.	1306
10	rehabilitation therapist*1.ti,ab.	78
11	occupational therapist*1.ti,ab.	3263
12	((speech or language) adj3 therapist*).ti,ab.	1218
13	1 or 2 or 3 or 4 or 5 or 6 or 7 or 8 or 9 or 10 or 11 or 12	251500
14	(mhealth* or m-health* or mobile health*).ti,ab.	80
15	(handheld adj3 (device* or computer* or technolog* or PC)).ti,ab.	360
16	(hand-held adj3 (device* or computer* or technolog* or PC)).ti,ab.	170
17	(tablet adj3 (device* or computer* or technolog* or PC)).ti,ab.	88
18	(slate adj3 tablet*).ti,ab.	1
19	(android adj3 tablet*).ti,ab.	1
20	(palm*1 adj3 (device* or computer* or pc)).ti,ab.	66
21	(pocket* adj3 (device* or computer* or pc)).ti,ab.	76
22	(mobile adj3 (computer* or PC)).ti,ab.	125
23	(portable adj3 (computer* or PC)).ti,ab.	88

24	(palm pilot* or palmpilot*).ti,ab.	39
25	(smartbook* or smart-book*).ti,ab.	0
26	ipad*.ti,ab.	33
27	i-pad*.ti,ab.	1
28	galaxy tab.ti,ab.	0
29	kindle fire.ti,ab.	0
30	playbook.ti,ab.	17
31	((touchscreen or touch screen) adj3 (computer* or PC or technolog* or device*)).ti,ab.	151
32	(smartphone* or smart phone*).ti,ab.	162
33	iphone*.ti,ab.	52
34	i-phone*.ti,ab.	8
35	(blackberry or black-berry).ti,ab.	24
36	(android adj3 (phone* or cellphone* or telephone* or device* or mobile*)).ti,ab.	4
37	(google adj3 (phone* or cellphone* or telephone* or device* or mobile*)).ti,ab.	2
38	(nexus one adj3 (phone* or cellphone* or telephone* or device* or mobile*)).ti,ab.	0
39	(google adj3 android).ti,ab.	2

40	(ipod touch or i-pod touch).ti,ab.	14
41	((touchscreen or touch screen) adj3 (phone* or cellphone* or telephone*)).ti,ab.	3
42	apps.ti.	11
43	or/14-42	1412
44	cellular phones/	1199
45	((cell or cellular) adj3 (phone* or telephone*)).ti,ab.	948
46	cellphone*.ti,ab.	36
47	(mobile adj3 (phone* or telephone* or device* or technolog*)).ti,ab.	1934
48	digital assistant*.ti,ab.	336
49	pda*.ti,ab.	726
50	44 or 45 or 46 or 47 or 48 or 49	3727
51	Internet/	18888
52	internet.ti,ab.	18585
53	(online or on-line).ti,ab.	27255
54	(web* or www).ti,ab.	24331
55	(wireless or wifi or wi-fi or wap).ti,ab.	757

56	(bluetooth or blue tooth).ti,ab.	31
57	51 or 52 or 53 or 54 or 55 or 56	59463
58	50 and 57	1006
59	43 or 58	2271
60	13 and 59	255
61	meta-analysis.tw.	12265
62	effectiveness.tw.	95254
63	search*.tw.	52624
64	61 or 62 or 63	154131
65	60 and 64	40
66	2012*.dp,yr,up.	182939
67	65 and 66	10
